# Bile Acids as a New Type of Steroid Hormones Regulating Nonspecific Energy Expenditure of the Body (Review)

**DOI:** 10.17691/stm2020.12.5.13

**Published:** 2020-10-28

**Authors:** P.P. Zagoskin, E.I. Erlykina

**Affiliations:** Associate Professor, Department of Biochemistry named after G.Ya. Gorodisskaya; Privolzhsky Research Medical University, 10/1 Minin and Pozharsky Square, Nizhny Novgorod, 603005, Russia; Professor, Head of the Department of Biochemistry named after G.Ya. Gorodisskaya Privolzhsky Research Medical University, 10/1 Minin and Pozharsky Square, Nizhny Novgorod, 603005, Russia

**Keywords:** bile acids, steroid hormones, obesity, cardiovascular diseases, regulation of the body energy metabolism

## Abstract

The review is devoted to the systematization, classification, and generalization of the results of modern scientific research on the role of bile acids as a new class of steroid hormones. The paper presents the evidence for bile acid participation in the regulation of the body energy metabolism, body weight control, as well as the pathogenesis of obesity, diabetes mellitus, insulin resistance, and cardiovascular diseases.

Particular attention is paid to the role of bile acids in the control of nonspecific energy expenditure of the body. The applied aspects of using the novel data about the membrane and intracellular receptors responsible for the development of hormonal regulatory effects of bile acids are analyzed. According to the authors, the modern data on the role of bile acids in the regulation of body functions allow a deeper understanding of the pathogenesis of body weight disorders and associated cardiovascular diseases. The review demonstrates promising directions in the search for specific methods of prevention and correction of these pathological conditions.

## Introduction

The investigation of the problem of body weight regulation is important for understanding the pathogenesis of cardiovascular, endocrine, and metabolic disorders associated with obesity. The obesity mechanism is a very complicated process. In particular, it includes disorders in the nonspecific expenditure of body energy as a fundamental element in the development of obesity. In recent years, an essential role of bile acids as regulators of energy metabolism has been found out. Bile acids in the intestines act as emulsifiers of dietary fats, activators of pancreatic lipase, and accelerators of the absorption of fat digestion products. However, when entering the blood vessels of the systemic circulation, they act as typical steroid hormones having specific intracellular receptors in the target cells.

The main bile acid receptors are farnesoid X receptor (FXR), pregnane X receptor (PXR), vitamin D receptor (VDR), glucagon-like peptide receptor (GLP-1), Takeda protein receptor 5 associated with G-protein (Takeda G protein-coupled receptor 5, TGR5), constitutive androstane receptor (CAR), and some others. The expression level of the genes encoding the receptors changes in a number of pathological conditions. The interaction of bile acids with these receptors causes various regulatory effects, the final stage of which is an increase in nonspecific energy expenditure of the body. This reduces the likelihood of developing obesity, diabetes, insulin resistance, liver steatosis, cardiovascular disease, and some other diseases. It is quite obvious that the sites for the synthesis of bile acids, their transport, interaction with the intestinal microbiota, as well as various receptors can serve as potential targets for the development of new therapeutic and preventive measures aimed at obesity and cardiovascular disease (CVD) management.

Cardiovascular diseases have been the number one cause of death in the world population for many years. Among them, the main ones are those with obesity as an essential element of the pathogenesis (atherosclerosis, hypertension, metabolic syndrome, etc.). Therefore, fighting obesity is the leading strategic goal of medicine aimed at reducing mortality [1–8].

The pathogenesis of obesity includes disturbances in the energy ratio between food calories and motor activity, neuroendocrine disorders, as well as changes in the body nonspecific energy expenditure. It is the latter aspect that presents the least studied and most promising area of medical science. With deeper study of nonspecific energy expenditure, there are great hopes for solving the problem of body weight regulation, prevention, and treatment of obesity and concomitant CVD and endocrine diseases [9].

Among the factors directly involved in the regulation of nonspecific energy expenditure of the body, bile acids are attracting more and more researchers’ attention. This information explosion was caused by the discovery of a new role of bile acids as typical steroid hormones with special receptors in many target cells of the body.

**The aim of this review** is to classify, summarize, and, if possible, describe in detail the regulatory effects of bile acids as a new type of steroid hormones that regulate body weight. The main task is to identify potential targets of the regulatory effects of bile acids, which can be used in developing new methods for the prevention and treatment of obesity and associated forms of CVD.

## Physicochemical properties and basic functions of bile acids

**Figure 1:**
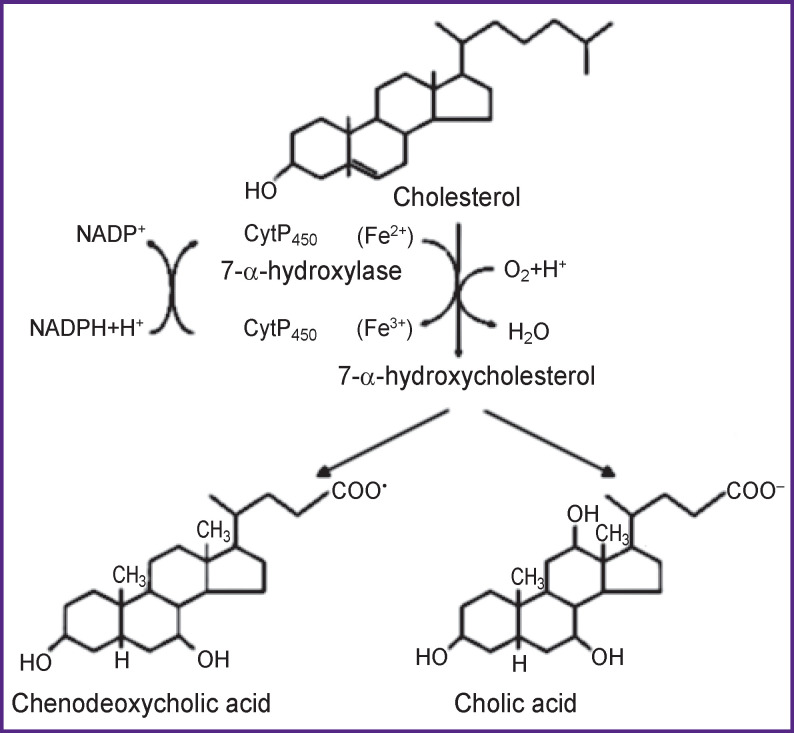
Synthesis of bile acids in the liver

Primary bile acids — cholic (CA) and chenodeoxycholic (CDCA) — are synthesized in the liver from cholesterol, and, therefore, they are typical steroids, such as sex hormones, gluco- and mineralocorticoids, vitamin D forms (Figure 1).

Oxidation of the cholesterol molecule with the introduction of oxygen atoms into the sterane ring and aliphatic radical leads to a sharp increase in the diphilicity of bile acids and appearance of powerful surfactant properties. Therefore, bile acids are the most effective emulsifiers of edible fat, stabilizers of colloidal micelles of fatty acids, cholesterol and 2-monoacylglycerols, and activators of pancreatic lipase.

Lipid digestion and absorption of digestion products are absolutely impossible without bile acids. These acids are the main chemical components of bile, responsible for all of its functions, including the elimination of free and esterified cholesterol, hydrophobic metabolites with a molecular weight of 300–500 Da, such as bilirubin and porphyrins. Besides, bile acids promote the elimination of many xenobiotics, medicinal compounds, and heavy metals [10, 11].

**Figure 2:**
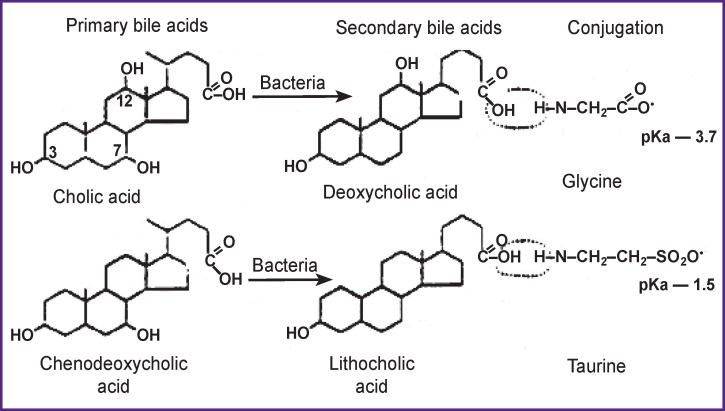
Conversion of bile acids in the enterohepatic cycle

In the process of performing all of these functions, bile acids circulate in the so-called enterohepatic cycle, which includes the liver, biliary tract, small intestine, and portal vein. This makes it possible to very sparingly maintain the pool of bile acids and provide the necessary level of secondary bile acid formation by their conjugation with glycine and taurine wherein glycocholic (GCA), taurocholic (TCA), as well as glycochenodeoxycholic (GCDCA) and taurochenodeoxycholic (TCDCA) acids are produced (Figure 2).

## Role of the intestinal microbiome in bile acid metabolism

The intestinal microbiome (gut microbiota) of humans and animals has evolved as a symbiotic part of the environment integrated with the host organism. The interaction of several trillion microbial bodies of the microbiome and the host body is in many ways mutually beneficial, although this coexistence is by no means always conflict-free. Intestinal microbes form a number of metabolites that are used by the host body as vitamins, hormones, immunostimulants, activators of intestinal motility, antibiotics that protect the intestines from pathogenic microflora. On the other hand, microbiota can synthesize a number of carcinogenic compounds, allergens, proinflammatory factors, and toxins, and also contribute to the development of diarrhea or intestinal constipation [12–14]. The gut microbiota performs its functions strictly synchronously with the functions of the macroorganism, and, therefore, its metabolism is an indispensable part of the host circadian rhythms [15–17].

Bile acids have the ability to regulate the optimal species composition of the microbiome both directly and indirectly, i.e. by activating the genes of the innate immune response in the small intestine [18]. For example, some primary bile acids, such as CA, TCA, and GCA, can stimulate the germination of *C. difficile* spores, but CDCA can prevent their germination [19]. Japanese researchers have recently proved the evidence of the inhibitory effect of bile acids against *Blautia coccoides and Bacteroides thetaiotaomicron* pathogenic species [20]. Disturbances in the normal intestinal microflora are correlated with the development of many forms of pathology, such as obesity, liver, and cardiovascular diseases [21–32]. Due to the constant contact of bile acids with the microbiome, part of the circulating bile acids is gradually reduced to their deoxy forms: deoxycholic (DCA), ursodeoxycholic (UDCA), and lithocholic (LCA) acids [33].

Bile acid metabolism includes chemical transformations occurring in the liver and intestines during the bile acid circulation in the enterohepatic cycle. Thus, a number of microorganisms in the small intestine catalyze the deconjugation of paired bile acids via the bile salt hydrolase (BSH) enzyme and subsequent dehydroxylation with the formation of unconjugated free bile acids and secondary bile acids, respectively [34].

In the human colon, CDCA is converted into UDCA. These acids differ only in the configuration of the hydroxyl group at C7 position (beta for UDCA and alpha for CDCA). However, UDCA is a hepatoprotective agent, while CDCA is a highly toxic substance [35].

Symbiotic microbes and the host immune system have evolved together for mutually beneficial regulation. On the one hand, the host bile acids can change the species composition of the microbiome and, on the other hand, the microbes are able to regulate the host immune system, in particular, by producing a number of their own metabolites. Some of these metabolites regulate the immune system via expression in the immune cells of the metabolite-specific receptors such as P2X7, GPR41, GPR43, GPR109A, the aryl hydrocarbon receptor (AhR) precursor, PXR, FXR, TGR5, and other molecular targets. The microbial metabolites and their receptors generate a vast array of signals that can respond to changes in the diet, health, and immunological status. As a consequence, the signals from the microbial metabolite promote nutrient uptake, regulate metabolism, and the host immune system. It is important that the microbial metabolites function bidirectionally, promoting both tolerance to certain food components and immunity so as to fight effectively the infectious microflora [36].

## Toxicological characteristics of bile acids

All biologically active derivatives of cholesterol become very dangerous with the excessive level of their physiological concentration in the blood and body tissues. Thus, steroid hormones have a carcinogenic effect. Colorectal tumors, breast, prostate, ovary cancer, and a number of others can be caused by overproduction or excessive administration of certain types of steroid hormones [37–41].

Vitamin D is a classic steroid with the highest toxicity among all vitamins if its concentration significantly exceeds the physiological level [42].

Bile acids are no exception, since they become very harmful substances if they are accumulated in the body in inadequately high concentrations, for example, when the bile ducts are blocked [43–47]. With gastric or esophageal reflux of bile acids, malignant degeneration of the stomach and esophagus cells may occur [48–50].

The cytotoxic effect of bile acids on hepatocytes, enterocytes, kidney canalocytes, and other cells is noted. Most likely, this effect is associated with the detergent action of bile acids on the membrane phospholipids and the activation of the cell death program [51–55]. Lithocholic acid is a secondary bile acid formed during the dehydroxylation of chenodeoxycholic acid by intestinal microbial enzymes. It is toxic and carcinogenic; therefore, it must be effectively neutralized in the liver [56–58].

## Bile acid cell receptors

In healthy people, the concentration of bile acids in the blood is very low and varies in a wide (but only in the micromolar) range, depending on the phase of digestion, age, sex, physiological status, etc. [59–61].

The level of bile acids in the blood increases sharply with the development of cholestasis, regardless of its origin [62, 63]. In these cases, the toxic properties of bile acids are displayed in full. The concentration of bile acids in hepatocytes of healthy people is 100–1000 times lower than in bile [64]. Thus, such a low concentration of bile acids in the blood and tissues excludes their carcinogenic, cytotoxic, or detergent effect. No doubt, the idea of the non-accidental presence of bile acids in the blood should arise as well as the assumption of the regulatory function of bile acids in the blood. This assumption was convincingly confirmed several years ago when previously unknown high-affinity bile acid receptors, FXR and TGR5, were discovered [65, 66].

It was after the discovery of the ability of specific cellular receptors to bind bile acids, that the latter were recognized as a new class of steroid hormones [67]. Moreover, it has become quite obvious that bile acids are the only type of steroid hormones (and perhaps the only type among all hormones) that have both intracellular and membrane receptors. Most likely, this is due to the high degree of diphilicity and surface activity of bile acids, which is in good accordance with their amazing ability to exist and diffuse both in the aqueous and lipid phase [68, 69]. Beside FXR and TGR5, VDR, CAR, PXR, and a number of others are also the receptors capable of interacting effectively with bile acids.

### Farnesoid X receptor.

 FXR was first detected in 1999 in the nuclei of the ileal enterocytes. Afterwards, the binding of bile acids to this receptor was found to activate the expression of the fibroblast growth factor gene (FGF 15/19), which, in turn, acting on hepatocytes, represses the synthesis of bile acids, gluconeogenesis, but activates the synthesis of proteins and glycogen [70]. Later, this receptor was detected in other body organs and tissues, in the cardiovascular system in particular [71]. While being a typical nuclear receptor, FXR, when bound to bile acids and/or their agonists, activates the transcription of specific genes in the DNA of target cells, which results in changing the metabolism and functions of these cells [72]. The metabolic effects of FXR, in addition to the liver and intestines, are required for the regulation of the functions of the cardiovascular system, kidneys, and pancreas [73]. Numerous studies in recent years have revealed the crucial importance of impaired FXR functions in obesity [74–77], CVD [78, 79], non-alcoholic fatty liver disease [80], metabolic syndrome [81], type 2 diabetes mellitus [82], and also other diseases. That is why FXR is a promising target for pharmacological studies on search for drug ligands capable of the targeted changing of the receptor expression and its regulatory activity [83].

### G-protein-coupled membrane receptor.

 Quite recently, in experiments on animals, and then in the study of human organ and tissue samples, new receptors have been identified that are capable of selectively binding bile acids as ligands. These membrane receptors, coupled with the functioning of a specific G protein, were named Takeda G protein-coupled receptor 5 (TGR5) in honor of the Japanese author who first described them [84].

TGR5 receptors are expressed with a high degree of intensity in the gallbladder, biliary epithelium, white and brown adipocytes, skeletal muscles, intestines, kidneys, placenta, and the brain [85].

The clinical studies conducted by an international team of researchers in 2013 on a large group of subjects showed that the TGR5 receptor gene is very active in the adipocytes of subcutaneous adipose tissue, and the expression level positively correlates with the development of obesity and decreases with weight loss when following a special diet [86]. TGR5 is a typical membrane receptor with its regulatory function mediated by a specific G protein. Bile acids are the main ligand of this receptor [87, 88]. Their binding to the recognition site of the receptor triggers a cAMP-dependent cascade mechanism for regulating the metabolism and functions of the target cells. The final cellular response depends on the type of target cells and the specific set of enzymes in them [89–91]. Not only bile acids, but also many other substances can be coactivators or inhibitors of this receptor [92–96]. Further studies of all stages of the events resulted from the interaction of these ligands with TGR5 have a great therapeutic potential [97].

### The role of glucagon-like protein in achieving the regulatory effects of bile acids.

 The regulatory action of bile acids can be mediated by hormones of the gastrointestinal tract (enterohormones). These hormones play a pivotal role in controlling nutrient metabolism and hold great promise for the treatment of type 2 diabetes and obesity. In particular, glucagon-like peptide-1 (GLP-1, incretin) promotes the release of insulin, inhibits glucagon secretion from the pancreas, reduces appetite and excess food intake, as well as gastrointestinal motility. GLP-1 is secreted by enteroendocrine L cells, which include ~1% of intestinal epithelial cells. L cells are scattered throughout the intestinal tract, their number increasing towards the distal small intestine and the initial section of the colon. These cells have TGR5 that can recognize and bind bile acids. The GLP-1 secretion and the performance of the regulatory effects described above are the physiological response of L cells to the addition of bile acids [98]. Soon, however, FXR activation by bile acids in L cells was shown to decrease GLP-1 secretion [99]. Presumably, the total response of L cells to the action of bile acids is supposed to depend on the ratio of both types of the receptors and the effectiveness of their action.

### Vitamin D receptor.

 Bile acids, as well as cholecalciferol, can also bind to VDR, since both of these ligands contain a sterane ring, the common structural details of which are recognized by this receptor [100]. VDR is expressed in many tissues and cells of the human body, such as the intestines, kidneys, β-cells of the islets of Langerhans, hepatocytes, osteoblasts, adipocytes, vascular smooth muscle cells, monocytes, and immunocompetent cells. VDR plays a central role in mineral homeostasis, the regulation of bone tissue metabolism, and is involved in the control of cell growth and differentiation. Calcitriol (1,25-dihydroxyvitamin D_3_), a steroid-like molecule with a partially destroyed steroid nucleus, and bile acids (LCA, but not CDCA and CA) are endogenous VDR ligands. A specific feature of VDR is that it can perform the function of both an intracellular and membrane receptor. When the ligand binds, VDR moves into the nucleus, where it binds to a specific region of DNA and modulates gene transcription. Genomic action is rather slow (hours) and includes downregulation of CYP7A1 expression, a specific isoform of cytochrome P450, and induction of the expression of its other isoform — CYP3A4, an enzyme that carries out oxidative detoxification of LCA. On the contrary, the response initiated on the membrane is rapid (minutes) and leads to the formation of signaling cascades which contribute to CYP7A1 repression in the liver. In the epithelium of the bile duct, VDR (together with FXR) was shown [101] to stimulate the production of antimicrobial proteins such as cathelicidins, which can supplement the immunomodulatory function of vitamin D.

### Constitutive androstane receptor.

 The androstane receptor CAR (NR1I3) has recently been described as a regulator of energy metabolism. It is a member of the nuclear receptor superfamily. However, CAR has some properties that distinguish it from many other nuclear receptors. First, its structural features allow it to exhibit constitutive activity in the absence of a ligand and interact in a species-specific way with a huge number of ligands that differ in chemical structure and origin. Second, this receptor is involved in the regulation of various physiological functions such as gluconeogenesis; metabolism of fatty acids, bilirubin, and bile acids; hormonal regulation, etc. Thirdly, a number of authors consider CAR as a xenosensor to the exposure of toxic substances by producing certain enzymes necessary to neutralize these toxins [102]. CAR regulates the expression of genes, proteins, and enzymes that act at all stages of liver metabolism and transport, including enzymes of phase I monooxygenase reactions, various types of phase II conjugation, and transport proteins involved in phase III [103].

The important role of CAR as a regulator of physiological processes has become evident partly due to the ability of this receptor to modulate the level of endogenous substances, including bile acids, thyroid hormones, hemes, and steroids. Besides, CAR activity has an impact on cholesterol homeostasis and signaling pathways that control food intake. This receptor also regulates many cellular processes, such as cell proliferation, inflammation, tissue damage and regeneration, immune response, and carcinogenesis [104].

Thus, the biological and toxicological processes regulated by this receptor are an integral part of whole-body health control. Together with the fundamental scientific interest, the study of CAR has an important medical aspect, since changes in its performance can cause pathology. On the other hand, searching for agonists and coactivators of this receptor enables to expand the possibilities in the therapy of the diseases associated with its dysfunction. Thus, the experiments on mice demonstrated that artificial activation of CAR protects the liver from damage caused by bile acids [105].

### Pregnane X receptor.

 PXR was originally identified as the “master” of xenobiotic sensing. It controls the expression of proteins involved in the transport, metabolism, and elimination of xenobiotics and a number of endogenous substances. Moreover, PXR has the function of regulating several signaling pathways associated with certain physiological processes controlled by bile acids. In particular, lithocholic acid and its 3-keto-derivative were found to activate human and mouse PXRs [106]. At the same time, 3-keto LCA is recognized as a more potent ligand of PXR than LCA, while CDCA, DCA, and CA only slightly activate PXR. Thus, PXR is recognized as the LCA receptor responsible for the detoxification of this hepatotoxic and potentially carcinogenic bile acid through the induction of its metabolic enzymes. PXR is produced primarily in the liver, intestines (ileum), and kidneys. This receptor regulates enterohepatic circulation and bile acid metabolism, as well as modulates liver regeneration, inflammation, and growth [107].

## Regulatory effects of bile acids as steroid hormones

**Figure 3:**
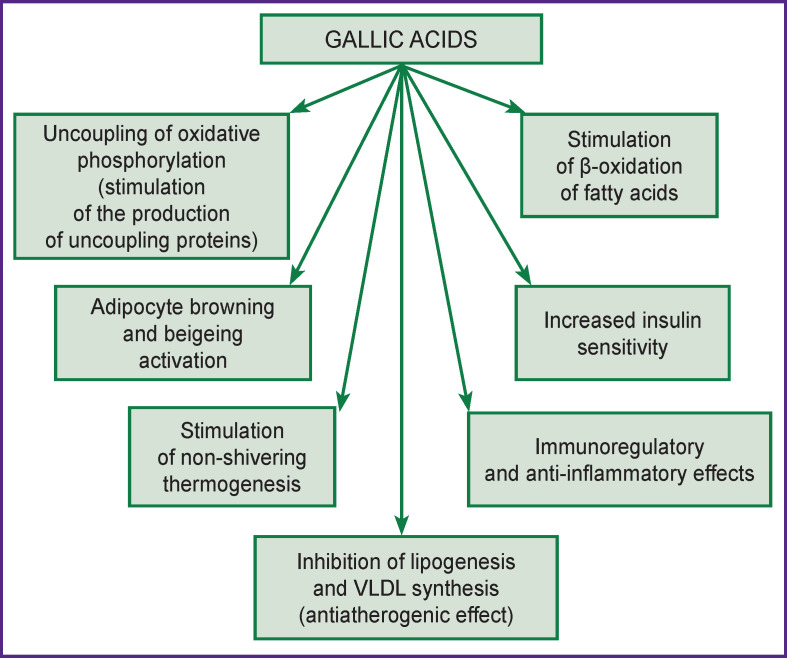
The main regulatory effects of bile acids

For a long time, bile acids were characterized only as participants in the process of digestion and absorption of lipids. At present, they are considered as a new type of metabolic modulators. Attached to various nuclear receptors of target cells, they form typical hormone-receptor complexes capable of activating the gene transcription of many proteins and enzymes involved in the regulation of a number of physiological processes that are in one way or another associated with body weight regulation and cardiovascular functions (extensive regulation). On some target cells, bile acids can be recognized and bound to membrane receptors. The formed hormone-receptor complexes trigger the mechanisms of cascade regulation of enzyme activity using G-proteins and intracellular messengers (intensive regulation). Bile acids as steroid hormones perform many functions of controlling energy homeostasis, carbohydrate, and lipid metabolism predominantly through the activation of nuclear FXR and cytoplasmic TGR5. The role of bile acids in the pathogenesis of the diseases such as obesity, diabetes mellitus, metabolic syndrome, and other “diseases of civilization” is becoming more elucidated [108]. The most typical responses of target cells to the regulatory action of bile acids as steroid hormones are shown in Figure 3.

### Uncoupling effect of bile acids.

 The direct uncoupling effect of bile acids on oxidative phosphorylation of mitochondria has long been known. It is associated with their powerful detergent effect on the phospholipid molecules of the lipid bilayer of biological membranes [109]. The uncoupling detergent effect of bile acids is usually observed *in vitro* in rather high, obviously non-physiological concentrations and, therefore, is not of great interest for practical medical use. However, the uncoupling effect of bile acids also occurs *in vivo*, and in much lower concentrations. This is not a direct but mediated effect through the formation of uncoupling proteins (UCP), regulation of non-shivering thermogenesis, and differentiation of preadipocytes to UCP-rich brown and beige cells. All these regulatory effects of bile acids contribute to an increase in nonspecific energy expenditure of the body, reduce the likelihood of developing obesity and associated CVD.

### Stimulating UCP protein production.

 Back in 2011, a group of scientists from several European countries proved that bile acids are regulators of energy metabolism. A protective effect against obesity caused by a high-fat diet, accumulation of lipids in the liver, an increase in triacylglycerols (TAGs) and plasma glucose levels was reported in mice that were orally administered bile acids. The authors showed that the plasma bile acid concentration in rats was increased by replacing the casein dietary protein source with salmon protein hydrolyzate (SPH). It is important to note that the rats that were given SPH were resistant to dietary obesity. They had decreased plasma glucose and TAG levels and decreased liver TAG content.

The increased concentration of bile acids in plasma is associated with the induction of genes for the proteins involved in energy metabolism and uncoupling of oxidative phosphorylation in the interscapular brown adipose tissue. It is interesting to note, that the same transcription pattern was found in white adipocytes of the subcutaneous adipose tissue and in the visceral fat. The rats fed with the SPH diet showed an increase in body energy expenditure and heat dissipation. The expression of the peroxisome proliferator-activated receptor beta/ delta, the uncoupling protein UCP3 and some other proteins increased in the skeletal muscles. The induction of the expression of the UCP3 protein gene in muscle by SPH was completely abolished by the incorporation of cholestyramine. All these data give evidence that bile acid metabolism can be modulated by diet and that this modulation can either prevent or mitigate the characteristic metabolic disorders caused by a high-fat diet [110].

Ziętak and Kozak [111] showed in experiments on mice that bile acids have their own thermogenic effect, inducing the synthesis of UCP1 in brown adipocytes, regardless of cold-activated sympathetic regulation. Cold exposure in animals and humans per se leads to the activation of the synthesis of bile acids due to the selective induction of the cytochrome P450-associated enzyme gene of oxysterol-7-alpha-hydroxylase (CYP7B1), a key enzyme in the synthesis of bile acids from cholesterol. The formed bile acids stimulate thermogenesis, mediated by the UCP regulatory influence. CYP7B1 deficiency leads to a decrease in thermogenesis, but, on the contrary, overexpression of this enzyme stimulates thermogenesis [112].

### Activation of browning and adipocyte beigeing.

 The transformation of preadipocytes into more differentiated cells — adipocytes (browning) is regulated by a number of cytokines and hormones, bile acids belonging to them. The color of these adipocytes depends on the presence of a large number of mitochondria which contain colored heme proteins — cytochromes. The cells containing fewer mitochondria and cytochromes are light brown (beige) in color, and the process itself of this differentiation is called beigeing. Beigeing is a corresponding transformation of adipocytes in white adipose tissue [113–115]. Cold effect on the body serves as a powerful signal for the stimulation of browning and adipocyte beigeing of [116].

At present, adaptive thermogenesis has been proved to be an energy-consuming process mediated by cold-activated browning and adipocyte beigeing and it is accompanied by an increased intake of carbohydrates and triglycerides delivered by LDL to these thermogenic cells. The mechanism of adaptive thermogenesis involves the induction of one of the enzymes of the cytochrome P450 class, family 7, subfamily b, which has a polypeptide chain 1 (CYP7B1). As mentioned above, this enzyme catalyzes the hydroxylation of cholesterol at position 7, which is the first step in the synthesis of bile acids. It results in the increase of their level in blood plasma, excretion with feces, specific changes in the intestinal microbiome occur, and, most importantly, heat production increases [117].

Bile acids produced by hepatocytes, stored in the gallbladder and released from it after ingestion, enter the intestines, are metabolized by the intestinal microbiota and are partially converted into secondary forms such as LCA and DCA. They, in turn, can activate FXR and TGR5 receptors responsible for the production of substances directly involved in the activation of thermogenesis [118].

All this gives grounds to conclude that bile acids are effective activators of non-specific energy expenditure of the body and direct regulators of body weight. Congenital or acquired disorders of the synthesis, metabolism, and reception of bile acids are involved in the pathogenesis of obesity and associated CVD.

### Bile acid stimulation of non-shivering thermogenesis through the activation of type 2 muscle iodothyronine deiodinase.

 In 2011, a group of scientists from the United States [119] found that mice with an experimentally induced defect in the thyroid hormone receptor-α (Thrα) gene developed a disturbance in cold resistance caused by undetected defects in the activation of the production of uncoupling proteins in brown adipose tissue (BAT). These mice developed an alternative form of facultative thermogenesis, which is activated when the ambient temperature decreases below the thermoneutrality values. In mice with Thrα-0/0, an increase in the amount of mRNA of the iodothyronine deiodinase type 2 enzyme (DIO2) in muscles and other tissues of the body was constantly observed. The authors showed that the activity of DIO2 increases in proportion to the increase in the mRNA level and is mediated by the sympathetic nervous system, as is noted in wild-type mice, but the effect of sympathetic regulation was more significant. In mice defective in the DIO2 gene (DIO2-/-), the authors showed that despite the differences in the severity of disturbances in thermogenesis in BAT in the mice with a defect in the α-receptor and in the mice with a defect in the UCP1 gene, they did not show an increase in oxygen consumption and they did not gain more weight than the wild-type control animals on a fat-rich diet. The Thrα-0/0 mice had increased levels of UCP3 mRNA, particularly, if they were on a fat-rich diet. It is important to note that the production of UCP3 mRNA is very sensitive to the action of thyroid hormones.

Besides, it was found [119] that the formation of muscle UCP3 mRNA in hypothyroid mice with Thrα-0/0 depends on the thyroid hormone level, which indicates the role of DIO2 that is responsible for the formation of triiodothyronine (T3). Finally, the authors found that bile acids stimulate not only BAT but also DIO2 activity in muscle tissue, and that the latter effect is associated with increased expression of muscle UCP3 mRNA, which depends on the level of thyroid hormones. These data provide a powerful argument in support of the concept that an increase in DIO2 activity in animals with Thrα plays a key role in alternative thermogenesis, the essence of which is an increase in fat oxidation due to increased local T3 generation in skeletal muscle [119].

A few years later, another group of researchers [120] gave evidence that DIO2 induction under the influence of bile acids is possible not only in muscle tissue, but also in BAT. In rodents, this tissue is activated by bile acids, which stimulate DIO2 production in BAT via the TGR5 receptor, which finally leads to an increase in oxygen consumption and non-specific energy expenditure. Moreover, the possibility of similar regulation of thermogenesis in humans was demonstrated. Thus, oral administration of CDCA to 12 healthy women for two days led to an increase in BAT activity. The level of whole-body energy expenditure was significantly increased under the influence of CDCA administration. The Treatment of cultured *in vitro* brown human adipocytes with CDCA or specific agonists of the TGR5 receptors increased the DIO2 expression and the degree of dissociation of oxidative phosphorylation. There were no these effects when white adipocytes were used as test objects. Thus, the possibility of using enzymes involved in the metabolism of bile acids as targets for the development of new tools for the control of nonspecific energy expenditure of the body can be considered proven [120].

### Activation of β-oxidation of fatty acids.

 According to some authors, the preventive effect of bile acids on obesity is associated not only with their effect on the differentiation and metabolism of brown adipocytes, but also on the metabolism of white adipose tissue [121]. Thus, exposing the 3T3-L1 white adipocytes cultured *in vitro* to CDCA or glucose, it was found that bile acids can switch the metabolic pathways of carbohydrates and lipids in the direction necessary for a preventive action. The NMR spectroscopic analysis of the metabolic pathways clearly showed an improvement in the metabolic status of the cells that more actively oxidize fatty acids by β-oxidation.

This result made it possible to formulate the concept that bile acid-induced metabolic changes in white and brown adipocytes are not completely dependent on neuroendocrine signaling, as previously assumed. Moreover, according to the authors, further study of the mechanisms underlying these effects will undoubtedly show interesting targets for clinical modulation [121].

### Insulin resistance.

 The causes of insulin resistance in obesity and type 2 diabetes are not limited to impaired insulin signaling only, but also involve complex interactions between different metabolic pathways. The analysis of large datasets from metabolomics and lipidomics has shed new light on the role of metabolites such as lipids, amino acids, and bile acids in altering insulin sensitivity. Metabolites can regulate insulin sensitivity both directly, modulating components of the insulin signaling pathway, for example, insulin receptor substrates (IRSs) or protein kinase B, and indirectly, altering the substrate flow through gluconeogenesis, lipogenesis, lipid oxidation, synthesis and degradation of proteins in the liver [122]. Bile acids are most directly involved in the regulation of carbohydrate and lipid metabolism. An increase in the synthesis of bile acids in the liver causes an insulin-like effect, expressed in inhibition of gluconeogenesis and stimulation of glycolysis. In addition, bile acids reduce the stress state of the endoplasmic reticulum (ER stress), a key event in the development of insulin resistance. Therefore, the regulatory function of bile acids should be considered as one of the factors that prevent the development of metabolic syndrome, obesity, and type 2 diabetes [123, 124].

### Immunoregulatory and anti-inflammatory effects.

 The main function of bile acids is associated with the digestion and absorption of dietary lipids and the regulation of cholesterol homeostasis. Moreover, they are important signaling molecules involved in the formation of the body’s immune responses. More recently, the experimental and clinical results [125] have shown that bile acids have a positive effect in cholestatic and inflammatory diseases. The activation of specific receptors by bile acids changes gene expression in many tissues. This leads to transformations not only in bile acid metabolism but also in glucose homeostasis, lipid and lipoprotein metabolism, the regulation of intestinal peristalsis and inflammation within the gut-liver axis, the growth of pathogenic microbes in the intestines, as well as nonspecific energy expenditure.

Bile acids are able to induce the synthesis of a number of protective proteins with antibacterial activity [126], interleukins [127, 128], anti-inflammatory cytokines [129], as well as reprogram pro-inflammatory macrophages into anti-inflammatory phenotypes [130].

The immunoregulatory role of bile acids is also associated with their modulatory activity towards bacterial lipopolysaccharides (endotoxins). The specific immunoregulatory role of bile acids is most clearly manifested in the regulation of innate immunity in various systemic inflammatory diseases, inflammatory bowel diseases, allergies, psoriasis, cholestasis, obesity, metabolic syndrome, alcoholic liver disease, and colon cancer [131, 132].

### Inhibition of lipogenesis and synthesis of very low-density lipoproteins.

 Bile acids, acting through FXR, prevent excess fat deposition not only by stimulating nonspecific energy expenditure of the body, but also by inhibiting lipid synthesis (lipogenesis) and their transport within VLDL [133, 134]. In humans, FXR activation by bile acids leads to increased expression of the peroxisome proliferator-activated receptor alpha (PPARα) which is the main regulator of fatty acid metabolism. In accordance with the mechanism, FXR activation can lead to the activation of lipolysis, an increase in the rate of fatty acid oxidation, and a decrease in the level of lipogenesis. The list of the FXR effects also includes its effect on lipoprotein metabolism. Thus, FXR was shown [135] to be able to reduce the transport of lipids with the aid of blood plasma lipoproteins by decreasing the expression of the ApoAI and ApoCII apoproteins. Moreover, FXR enhances the expression of VLDL receptors, which contributes to cleansing the blood of “bad” cholesterol.

Resin substances, called bile acid sequestrants, reduce LDL cholesterol levels by 10–30% by reducing the absorption of bile acids from the intestines, which reduces the pool of bile acids circulating in the enterohepatic cycle. This, in turn, causes the activation of the synthesis of bile acids from cholesterol, a decrease in its level in the liver and blood plasma [136].

## Changes in bile acids and their receptors in obesity and cardiovascular disease

The disorders of bile acid metabolism and expression of their specific receptors lead to energy imbalance and progressive obesity. In this case, obesity is the main risk factor for the development of atherosclerosis, type 2 diabetes, hypertension, dyslipidemia, and other concomitant forms of cardiovascular and endocrine pathology [137]. The causes of the “epidemic” of obesity remain unclear, although there are several hypotheses, including increased food availability, adaptation to a sedentary lifestyle, changes in food composition or nutritional value, intestinal dysbiosis, viral infection, low or high birth weight, evolutionary pressure or all taken together.

The history of the discovery of drugs for obesity is full of failures, except in cases of rare monogenic disorders, such as leptin deficiency, in which hormone replacement is effective. The pharmacological therapy aimed at regulating energy intake can curb appetite or reduce food cravings to some extent, but this often leads to significant and unacceptable cognitive or mental side effects.

The study of the hormonal function of bile acids and their receptors provides new possibilities for solving these problems. Various *in vivo* studies and clinical trials have shown significant beneficial effects of bile acids in reducing body weight, restoring insulin sensitivity, and improving cardiovascular function [138].

In obesity, the normal binding of bile acids to various types of receptors (FXR, TGR5, CAR, PXR, etc.) is disrupted. Therefore, the control of the expression of bile acid receptors and the search for agonists and antagonists of these receptors provide conditions for the development of new pharmacological agents for the prevention and treatment of obesity, diabetes mellitus, and CVD [139].

## Conclusion

The analysis of the current scientific literature on studying the metabolism and regulatory role of bile acids leads to the conclusion about a new type of steroid hormones that regulate body energy homeostasis, body weight, and insulin sensitivity. The disorders of bile acid metabolism and expression of their specific receptors lead to energy imbalance and progressive obesity. Obesity, in turn, is the most common risk factor for the development of atherosclerosis and type 2 diabetes mellitus, followed by vascular disorders of all organs and tissues of the body. Therefore, the following recommendations are proposed as new measures for the prevention of atherosclerosis and cardiovascular pathology, quite obviously arising from the materials presented in this review:

1. mandatory monitoring of the hepatobiliary system in persons with a tendency to develop obesity (BMI >30);
2. regular assessment of bile acid levels, the ratio of their primary and secondary forms in the duodenal contents and blood in persons with initial forms of obesity;
3. mandatory monitoring of the intestinal microbiome in all persons included in the obesity risk group, as well as in patients diagnosed with obesity. In case of detecting intestinal dysbiosis, it is necessary to restore normal intestinal microbiota;
4. the use of bile sequestrants as a universal palliative method for lowering the bile acid level in the intestines when manifestations of cholestasis are detected [140–143];
5. further development of the methods for determining the levels of expression of the main bile acid receptors in order to put the knowledge of bile acids into clinical practice;
6. the study of the levels of nonspecific energy expenditure of the body and their changes due to disturbances of the regulatory function of bile acids, it being a particularly important factor in the prevention and subsequent correction of obesity.
